# Salmagundi

**Published:** 1887-12

**Authors:** 


### SALMAGUNDI.

EDITED BY E. G. BETTY, D.D.S.

Obituary.—Dr. Walter A. Dun died November 7th, at his residence No. 103 East Third street, Cincinnati. His disease, which was not the prevailing typhoid, but malarial remittent fever, complicated by meningitis, or brain fever in popular phrase, lasted four weeks. He was conscious the day before his death, for a little while, and knew his mother, who has been here for several days. The fight for life was a strong one, backed by youth, good habits and a strong frame. Dr. B. M. Rickets, Dr. Carson and Dr. Mackenzie have been the physicians in direct attendance, while the entire regular profession, notably Dr. L. A. Langdon and Dr. A. E. Heighway, jr., have been eager to do what they could.

Dr. Walter Angus Dun was born on his father's place "Plumwood," near London, Madison County, Ohio, March 1, 1857. His father and mother were Robert George and Anna L. Dun. His early education was by private tutor at home. At the age of sixteen he entered the University of Ohio at Columbus, where he graduated at nineteen with the degree of Bachelor of Science, in 1878. He began medical study at once, entering the Miami Medical College, Cincinnati, in the fall of 1879, and taking a three years' course. He finished at the head of his class, taking the Faculty prize. He successfully competed for the position of Interne, and as such spent the usual year in the Cincinnati Hospital. The following year was passed in study among the London hospitals, and he took the degree of Licenciate of the Royal College of Physicians, and became a member of the Royal College of Surgeons, on October 26, 1882, and November 15, 1882, respectively. He began practice in Cincinnati in 1883, declining the proffered position of resident physician to Girard College, Philadelphia, but accepting that of Trustee of Miami University, offered by Governor Hoadly. Since then, his career has been a wonderfully active one, though exceptionally interrupted by acute illness. He had typhoid fever when a medical student and since, has weathered attacks of measels, variola, and scarletina. His ambition was great, and his capacity for work must have been enormous. In spite of a large and growing practice and a fondness for social life, in which his rare personal qualities made him a favorite, he was at the time of his death occupying a clinical chair in the Miami Medical College, to accept which he resigned the charge of the microscopic and physiological laboratory of the same college. He was a member of the Executive Board of the Cincinnati Society of Natural History, of which he was President in 1886. He was a member of the staff of the Episcopal Hospital for Invalid Children ; a member of the American Association for the Advancement of Science ; of the American Medical Association ; of the Ohio State Medical Society ; of the Cincinnati Medical Society ; of the Cuvier Club ; of the Gymnasium ; of the Literary Club ; of the University Club ; of Douglass Lodge, K. of P.; of N. C. Harmony Lodge, No. 2, F. and A. M., and of the Police Examining Board. His contributions to natural history literature were frequent and able, his favorite departments being geology, zoology, and archæology. A recent article on the results of the physical examination of a thousand applicants from civil life for the Cincinnati Police is recognized as a rare and valuable contribution to medical literature.

In 1884 he delivered a series of lectures before the University of Cincinnati on "Comparative Anatomy and Physiology." He was also trustee of the estate of his father, who has been a paralytic since 1885.

Dr. Dun was an only son, and the first of a large family to die, his five sisters surviving. These are Mrs. S. J. Patterson, of Dayton, O.; Mrs. Edward Denmead, of Columbus, and the three Misses Dun. Deceased was s nephew of ex-U. S. Senator A. G. Thurman. He was noted for careful reflective qualities, cool discrimination, and conservative judgment. It is long since so promising a life has been so early cut off, and the mourners are numbered in every circle of city life.

The Natural History and other bodies will meet to-day to take approprite action. The medical profession will meet to-day at 3 P. M.

At a meeting of the Academy of Medicine, last night, the following tribute of respect to the memory of Dr. Dun was adopted by unanimous vote:

"The Academy of Medicine learns with great regret of the decease of one of its fellows, Dr. Walter A. Dun.

"Dr. Dun was an ardent follower of his profession, a man of high gifts, and one whose early life was a promise of a distinguished career. His devotion was not confined solely to the art which he practiced, but extended to the allied sciences of medicine. In him we lose not alone an active worker in our profession, but also a gentleman whose high type of character impressed every one who came within his presence—one in whom the manly virtues of truth, courage, and courtesy found their highest exemplification. The heartfelt sympathy of this Academy is extended to his family.

"W. E. KEILY,

"GEO. W. RYAN,

"WM. JUDKINS,

"Committee."

*Cin. Com. Gaz. Nov. 8th* 1887.

